# 

**Published:** 1884-06

**Authors:** 


					﻿Whole Number,
rcLxv.
jI0NE, 1884.
Number i i,
VolumeXX4IK-
THE
BUFFALO
Medical and Surgical Journal.
EDITORS :
THOS. LOTHROP, M.
A. R. DAVIDSON, M.
I». W. VAN PEYMA, M. D.
Published by the Medical Journal Association.
No. 3 WEST CHIPPEWA ST.
Entered at the Post-Office at Buffalo, N. Y., as Second-class Mail Matter.
				

